# Hematological parameters of bronchopulmonary dysplasia in preterm infants: a meta-analysis

**DOI:** 10.3389/fped.2025.1657314

**Published:** 2025-11-17

**Authors:** Hongyu Li, Jia Fan, Hehua Du, Jihong Pan

**Affiliations:** 1 Department of Neonatology Nursing, West China Second University Hospital, Sichuan University, Chengdu, Sichuan, China; 2 Key Laboratory of Birth Defects and Related Diseases of Women and Children (Sichuan University), Ministry of Education, Chengdu, Sichuan, China; 3 Institute of Sports Medicine and Health, Chengdu Sport University, Chengdu, Sichuan, China

**Keywords:** bronchopulmonary dysplasia, lung dysplasia, platelets, meta-analysis, preterm infants

## Abstract

**Background:**

Bronchopulmonary dysplasia (BPD) represents the most widespread and severe form of chronic pulmonary disease in preterm infants. Studies have revealed an association between BPD and hematological parameters (HPs); however, the findings are inconsistent.

**Objectives:**

This study utilized a systematic review and meta-analysis to summarize the association.

**Methods:**

The Web of Science, Cochrane Library, Embase, and PubMed were retrieved up to May 4, 2024, with an update on April 10, 2025. Studies investigating the correlation between HPs and BPD in preterm infants were included. This study adhered to the Preferred Reporting Items for Systematic Reviews and Meta-Analyses (PRISMA) guidelines. The quality of the studies was evaluated via the Newcastle-Ottawa Scale. Stata 18 and MetaDisc were utilized for statistical analysis.

**Results:**

This meta-analysis encompassed 20 studies with a total of 4,752 participants and investigated the association between HPs and BPD in neonates. Statistically significant differences were found between BPD and non-BPD infants for eight parameters: neutrophil (*N*EU) count, hemoglobin (HGB), monocyte count, hematocrit (HCT), neutrophil-to-lymphocyte ratio (NLR), red blood cell (RBC) count, platelet (PLT) count, and systemic inflammatory response index (SIRI). Further correlation analysis and effect size evaluation revealed that, while some parameters differed between groups, the association between key parameters and BPD risk was not significant. Specifically, the odds ratios (ORs) for HCT (OR = 1.33, 95% confidence interval [CI]: −0.11 to 2.77), PLT (OR = 1.0, 95% CI: 0.98–1.03), and HGB (OR = 1.38, 95% CI: 0.42–4.49) did not reach statistical significance, suggesting these three parameters may not be independent influencing factors for BPD risk. Concurrently, diagnostic performance analysis demonstrated limited discriminatory ability for NLR, with a receiver operating characteristic-area under the curve (ROC-AUC) of 0.670 (standard error [SE] = 0.054), and for PLT, with an ROC-AUC of 0.675 (SE = 0.067).

**Conclusions:**

The current study indicated significant differences in NEU, HGB, HCT, RBC, PLT, NLR, and SIRI between BPD and non-BPD patients, with elevated NEU, NLR, and SIRI and reduced HGB, HCT, RBC, and PLT. However, this study had its limitations. Further analysis requires more multicenter, large-sample prospective studies.

**Systematic Review Registration:**

https://www.crd.york.ac.uk/PROSPERO/, identifier CRD42024552716.

## 1 Introduction

Bronchopulmonary dysplasia (BPD) is the most common and serious chronic lung disorder affecting premature infants (1). The incidence of BPD ranges from 10% to 89% in extremely preterm infants (gestational age [GA] <28 weeks) worldwide, including 10%–73% in Europe, 30%–62% in Oceania, 18%–89% in North America, and 18%–82% in Asia (2). Furthermore, about 40% of infants with an ultra-low birth weight (less than 1,000 g) develop BPD (3). Although various therapies for BPD have significantly improved survival in neonates born before 28 weeks' GA, including surfactant therapy (4), postnatal steroid therapy (5), limiting the duration of invasive mechanical ventilation (6), and improved ventilation methods (e.g., noninvasive positive pressure ventilation [NIPPV] (7) or high-frequency oscillatory ventilation [HFOV]) (8), the incidence of BPD remains high. Studies have indicated that various factors, including the duration of mechanical ventilation, respiratory distress syndrome (RDS), early-onset sepsis, small for GA (SGA), patent ductus arteriosus (PDA), NLR, thrombocytopenia, and anemia, may contribute to the development of injury to the immature lungs, potentially leading to BPD. Nevertheless, the exact cause of BPD remains unclear, and effective prevention strategies are limited. Therefore, early identification and prevention of BPD are particularly important.

Current evidence suggests that preterm infants exhibit heightened pulmonary sensitivity to inflammatory stimuli due to immature alveolar development and incomplete pulmonary vascular bed structure. A persistent proinflammatory response, characterized by the infiltration of inflammatory mediators such as elastase and reactive oxygen species (ROS) released by neutrophils, can induce alveolar epithelial cell injury and pulmonary fibrosis. This ultimately culminates in BPD (9). Hematologic parameters (HPs), including neutrophil (NEU) count, neutrophil-to-lymphocyte ratio (NLR), and systemic inflammatory response index (SIRI), serve as ‘window biomarkers' that reflect systemic inflammatory status. This provides a pathophysiological basis for indirectly assessing pulmonary inflammatory imbalance via peripheral blood. Meanwhile, the current clinical practice for early BPD diagnosis relies heavily on imaging and respiratory support evaluation, lacking convenient, dynamically monitorable biomarkers. This unmet clinical need underscores the value of systematically investigating the association between HPs and BPD. Such research could facilitate the early identification of high-risk infants and inform targeted anti-inflammatory intervention strategies.

In recent years, several studies have examined the relationship between HPs and BPD. For instance, Chen et al. revealed a significant association between platelet (PLT) count and BPD in 115 preterm infants. The same study suggested that inhibiting PLT activation could relieve lung inflammation in extremely preterm infants (10). Duan et al.'s study on 149 preterm infants indicated that hemoglobin (HGB) levels ≤155 g/L during the initial three days of life were linked to an elevated risk of BPD (11). The researchers hypothesized that, during the transition from fetal to early neonatal life environment, HGB plays a significant role in adapting lung function to the new environment. Lower HGB levels may impair normal lung development and contribute to the development of BPD. Despite extensive studies on the association of HPs (such as NLR, PLT, HGB, and white blood cell [WBC] count) with BPD, the results are highly heterogeneous, and there is a lack of systematic reviews to comprehensively assess these parameters. Furthermore, the potential role of novel inflammatory composite indicators such as the monocyte-to-lymphocyte ratio (MLR) and the SIRI in BPD remains to be fully elucidated.

According to the present state of research, no study has systematically summarized and analyzed the use of HPs in BPD. Therefore, the current study adopted an evidence-based approach to evaluate the relationship between HPs and BPD via a meta-analysis that incorporated all relevant studies. The goal was to provide a reference basis for clinical treatment and prognosis.

## 2 Methods

### 2.1 Registration

This study's report was consistent with the Preferred Reporting Items for Systematic Reviews and Meta-analyses (PRISMA) guidelines (12). The study was also registered with the International Prospective Register of Systematic Reviews (PROSPERO) under the registration number CRD42024552716.

### 2.2 Data sources and search strategies

Four databases, including the Cochrane Library, Web of Science, PubMed, and Embase, were retrieved up to May 4, 2024, with no restrictions on language or publication date. The search was updated on April 10, 2025, to ensure timeliness. The search terms included “bronchopulmonary dysplasia”, “hematological parameters”, “platelet count”, “hemoglobin”, “white blood cell count”, and “hematocrit”. The specific strategy is described in Supplementary Table S1. To prevent omission, the reference lists of all relevant studies were also reviewed for additional publications.

### 2.3 Eligibility criteria

The eligibility criteria for the current study adhered strictly to the PECOS principles.

The following criteria were employed to include studies:

a. Population: The study population comprised neonates diagnosed with BPD using the following diagnostic criteria specified in the primary studies: the 2001 National Institute of Child Health and Human Development (NICHD) criteria, defining BPD as the need for oxygen or respiratory support at 28 days postnatal age (13); the 2018 NICHD criteria (14); and the 2019 Neonatal Research Network (NRN) definition, requiring oxygen or respiratory support at a postmenstrual age (PMA) of 36 weeks (15);
b. Exposure factors: all studied HPs, including WBC, NEU, MLR, HGB, lymphocytes, monocytes, hematocrit (HCT), red blood cell (RBC), PLT, NLR, platelet-to-lymphocyte ratio (PLR), mean platelet volume (MPV), red cell distribution width (RDW), platelet distribution width (PDW), C-reactive protein (CRP), SIRI;
c. Control: Non-BPD neonates;
d. Outcome indicators: Any of the following outcomes reported: i. Differences in HPs levels between the two groups; ii. Correlation data between HPs and outcomes; iii. Predictive data (e.g., specificity, sensitivity, or true-positive, false-positive, true-negative, false-negative), which can be utilized to calculate the diagnostic efficacy of HPs in relation to the outcomes;
e. Study type: Cohort or case-control study.

The following criteria were employed to exclude studies:

a. Conference abstracts, clinical trial registries, reviews, systematic reviews, meta-analyses, guidelines, animal experiments, and case reports;
b. BPD patients not involved;
c. Required HPs not covered;
d. Outcome indicators cannot be extracted or calculated;
e. Non-English articles.

### 2.4 Study selection

Two researchers (LHY and PJH) screened the articles based on eligibility criteria. After importing all relevant articles into EndNote X9 and removing duplicates, the rest were reviewed by titles and abstracts. The full texts of initially eligible articles were searched and reviewed to exclude ineligible studies. Any discrepancies were addressed via discussion, with unresolved issues adjudicated by a third researcher (DHH).

### 2.5 Extraction of data

Data were extracted via a predefined Microsoft Excel sheet. Two researchers (LHY and PJH) conducted the extraction independently and verified their respective results. Any discrepancies were settled via discussion, with unresolved issues adjudicated by a third researcher (DHH). The retrieved data included: (a) basic information: title, publication year, authors' names, population, country of study, source institution, and study type; (b) characteristics of included population: GA and weight of preterm infants at birth; number of BPD and non-BPD patients; weight, GA, and sex of BPD patients; and mother's gestational status; (c) type of HPs; (d) statistical methodology and outcome indicators.

### 2.6 Bias risk and rank certainty assessment

This meta-analysis included both cohort and case-control studies. Two researchers (LHY and PJH) evaluated the risk of bias (ROB) via the Newcastle-Ottawa Scale (NOS). The NOS comprises three domains: outcome, comparability, and selection (16). Any disagreements were resolved through discussion. Inter-rater agreement was evaluated using Cohen's kappa statistic, yielding a kappa coefficient exceeding 0.8. This indicates substantial consistency. All included studies had an NOS score of ≥6, indicating high quality (Table 1).

**Table 1:** The critical characteristics of enrolled studies in systematic review and meta-analysis.

No.	Author	Year of publication	Country	Study type	Patient source	Gestational age of premature infants	Number of cases: BPD/non-BPD	BPD				Hematological parameters	Outcome measures	NOS
								Diagnostic criteria	Weight	GA (weeks)	Gender (male/female)			
1	Wang	2023	China	Case-control	Single center	GA <32 weeks	134 + 162	2018 NICHD	1,120 ± 270	28.4 ± 1.33	80/54	HCT, WBC, N, Monocytes, Lymphocytes, HGB, PLT, RBC	A, B, C	7
2	Maytasari	2022	Indonesia	Cohort study	Single center	GA <36 weeks	22 + 94	2018 NICHD	NA	NA	NA	PLT, HCT	B	7
3	Jiang	2023	China	Case-control	Single center	GA <32 weeks	48 + 76	2001 NICHD	1,222 ± 283	28.7 ± 1.9	23/25	MPV, NLR, PLR, PMI, PLT, WBC, N, Lymphocytes, PDW	A, B, C	7
4	Ceran	2022	Türkiye	Case-control	Single center	GA <32 weeks	38 + 36	2001 NICHD	1,086 ± 316	28 ± 1.8	21/17	WBC, CRP	A, B, C	6
5	Cao	2023	China	Case-control	Single center	GA <32 weeks	50 + 72	2018 NICHD	1,251 ± 294	30.29 ± 1.67	24/26	MLR, SIRI, PLR, NLR, PLT	A	7
6	Cakir	2023	Türkiye	Case-control	Single center	GA <32 weeks	189 + 957	2001 NICHD	982 ± 201	27.6 ± 1.1	106/83	N, MLR, SIRI, Monocytes, Lymphocytes, PLT, PLR, NLR, CRP	A, B, C	7
7	Wang	2022	China	Case-control	Single center	GA ≤ 30 weeks	64 + 70	2001 NICHD	1,136 ± 194	28 ± 3.7	34/30	PLT, MPV, PDW	B, C	7
8	Go	2021	Japan	Case-control	Single center	GA ≤30 weeks	85 + 91	2001 NICHD	646 ± 157	24.9 ± 1.6	44/41	RDW, HGB	A, B, C	7
9	Sun	2019	China	Case-control	Single center	GA <32 weeks	144 + 152	2001 NICHD	1,252.47 ± 296.70	28.87 ± 1.54	81/63	N, NLR, Lymphocytes	A, C	7
10	Chen	2019	China	Case-control	Single center	GA ≤28 weeks	60 + 55	2001 NICHD	770 ± 165.2	25.8 ± 1.7	39/21	PLT, HCT, HGB, MPV, PDW	A, B, C	7
11	Duan	2016	China	Case-control	Single center	GA <32 weeks	61 + 86	2001 NICHD	1,184.51 ± 214.94	28.54 ± 1.42	43/18	HGB	A, B, C	6
12	Yang	2014	Korea	Case-control	Single center	GA <34 weeks	66 + 195	2001 NICHD	NA	NA	NA	PLT	A, B, C	7
13	wang	2022	china	Case-control	Single center	GA ≤32 weeks	40 + 42	2001 NICHD	1,353 ± 198.18	29.96 ± 1.10	25/15	WBC, N, Lymphocytes, HCT, HGB, PLT, RBC	A, B, C	7
14	Hee	2022	Korea	Case-control	Single center	GA < 30weeks	64 + 44	2001 NICHD	NA	NA	NA	RDW	A	6
15	Hellström	2021	Sweden	Case-control	Single center	GA < 30 weeks	213 + 239	2018 NICHD	NA	NA	NA	HGB, HbF	A, B	7
16	Inoue	2014	Japan	Case-control	Single center	GA < 31 weeks	16 + 52	2001 NICHD	707 ± 241.5	24.8 ± 1.85	NA	WBC, N, Monocytes, Lymphocytes	A, B	7
17	Marom	2016	Israel	Case-control	Single center	GA < 32 weeks	39 + 39	2001 NICHD	854 ± 174	27 ± 1.6	20 + 19	Lymphocyte PLT, N	A	7
18	Gao	2023	China	Cohort control	Single center	GA < 32 weeks	63 + 129	2001 NICHD	1,160 ± 230	NA	36 + 27	HGB, WBC, PLT	A, B	7
19	Tang	2024	China	Case-control	Single center	GA <32weeks	29 + 245	2001 NICHD	987.10 ± 220.64	28.67 ± 1.62	19 + 12	Lymphocyte, N, WBC, HGB, HCT, PLT	B	7
20	An	2025	China	Case-control	Single center	GA <32weeks	56 + 435	2019 NRN	1,019.54 ± 222.57	27.83 ± 1.80	30 + 26	N, PLT, Monocytes, Lymphocytes, SIRI	C	7

BPD, Bronchopulmonary dysplasia; GA, Gestational age (weeks); A, difference in hematological parameters between BPD and non-BPD; B, Correlation between hematological parameters and BPD; C, Predictive value of hematological parameters for BPD; NICHD, National Institute of Child Health and Human Development; NRN, neonatal research network.

### 2.7 Statistical analysis

Primary outcome indicators included WBC, NEU, MLR, HGB, lymphocytes, monocytes, HCT, RBC, PLT, NLR, PLR, MPV, RDW, PDW, CRP, and SIRI in BPD and non-BPD patients. Secondary outcome indicators were the correlation analysis of PLT, HGB, and HCT with BPD and the predictive value of PLT and NLR in BPD.

Statistical heterogeneity was quantified via Cochran's *Q* test and Higgins' *I*^2^. *P* < 0.1 or *I*^2^ > 50% indicated significant heterogeneity, in which case a random-effects model (REM) was employed. Otherwise, a fixed-effects model was utilized. In case of high heterogeneity, subgroup and meta-regression analyses were performed to identify the source. For analyses of differences in HPs levels between the two groups and the associations between HPs and BPD, a meta-analysis was conducted via Stata 18.0. Weighted mean differences (WMD) and 95% confidence intervals (CIs) were computed to assess possible differences in WBC, NEU, MLR, HGB, lymphocytes, monocytes, HCT, RBC, PLT, NLR, PLR, MPV, RDW, PDW, CRP, SIRI, between the BPD and non-BPD groups. Furthermore, 95% CIs and odds ratios (ORs) were calculated to evaluate the correlation between HPs and BPD. *P* < 0.05 indicated statistical significance for the pooled statistics of included studies. A diagnostic meta-analysis was conducted via Meta-Disc 1.4 and Stata software (version 18.0, Stata Corporation, TX, USA). The MIDAS module of the bivariate mixed-effects model was adopted for the analysis. Pooled sensitivity, specificity, diagnostic score (DS), diagnostic odds ratio (DOR), negative likelihood ratio (LR-), and positive likelihood ratio (LR+) were calculated via forest plots. Higher DOR and DS values were linked to better diagnostic performance. The area under the curve (AUC) was obtained by plotting the summarized receiver operating characteristic (SROC) curve. The diagnostic performance was low, medium, or high when the AUC was 0.5–0.7, 0.7–0.9, or 0.9–1.0, respectively. Deeks' funnel plot was drawn to assess potential publication bias in the diagnostic outcomes across studies. A *p*-value greater than 0.05 suggested a lack of substantial publication bias. To investigate potential publication bias in the correlation analysis, we generated funnel plots and performed an Egger test. When significant publication bias was detected and the number of studies exceeded ten, the trim-and-fill method was implemented to assess the impact of the bias on the results.

## 3 Results

### 3.1 Article search and study selection

**Figure 1:**
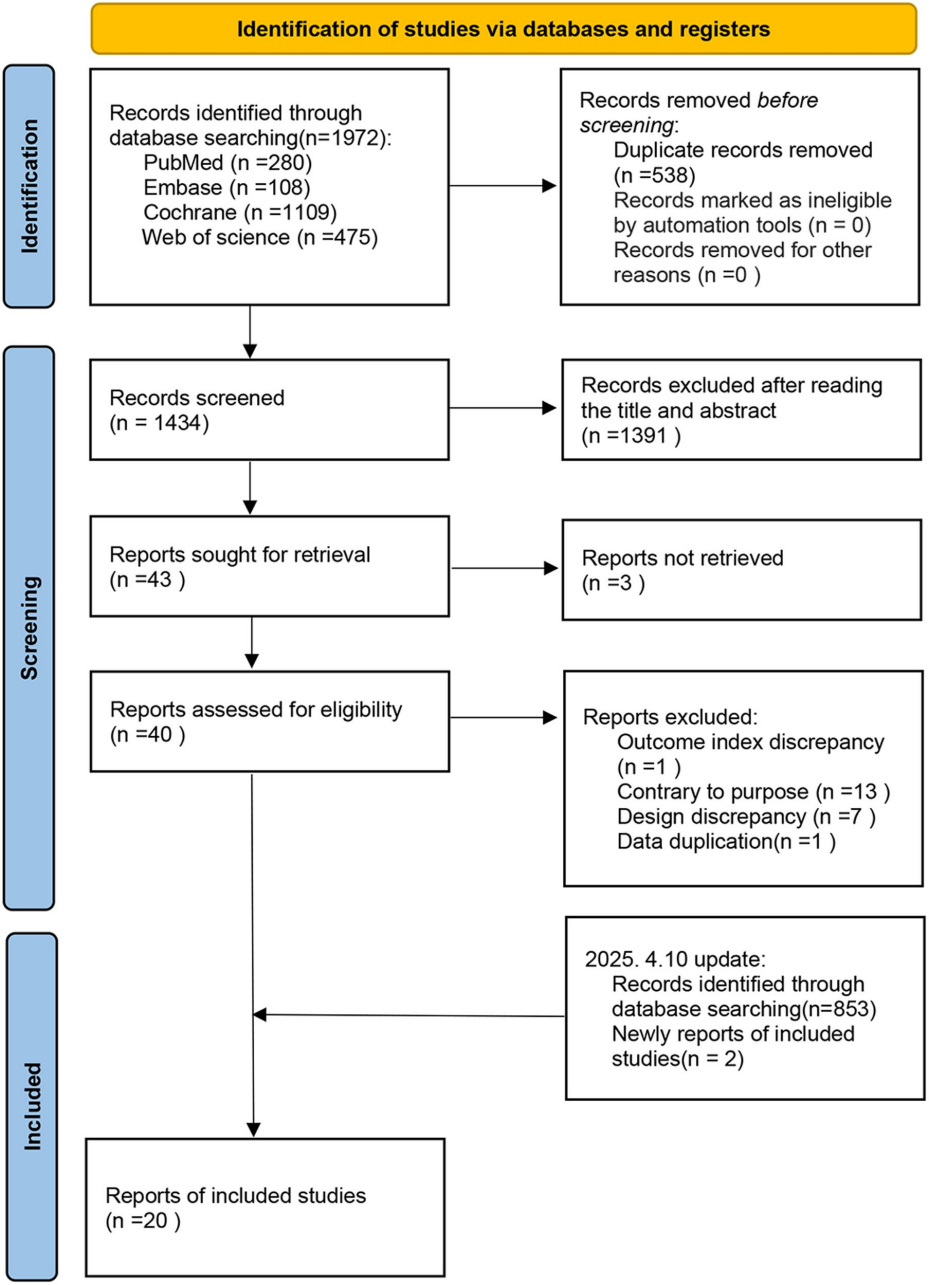
Search strategy.

Initially, 1,972 articles were identified. After removing 538 duplicates, 1,434 articles were screened by title and abstract, excluding 1,391 records. The full texts of the remaining 43 articles were reviewed, excluding 3 for unavailable full texts, 21 for ineligibility, and 1 for duplication. Ultimately, 18 eligible articles were selected. In addition, an updated search on April 10, 2025, yielded 853 new records, and 2 additional eligible studies were found. Finally, 20 articles were included in this meta-analysis, as illustrated in Figure 1.

### 3.2 Characteristics of included studies

Twenty studies (10, 11, 17–34) were included in this meta-analysis. The analysis involved 4,752 participants, of whom 1,554 were BPD patients and 3,198 were non-BPD patients. Seven countries were involved, i.e., China, Indonesia, Turkey, Japan, South Korea, Sweden, and Israel. All the studies included were single-center studies, encompassing 18 case-control and 2 cohort studies. The mean weight of BPD patients was 646–1,353 g, the mean GA was 24.9–30.29 weeks, and there were more males than females. For differences in HPs with respect to BPD, seven studies examined WBC (18, 20, 25, 26, 29, 30, 34), 9 examined NEU (18, 20, 21, 24–26, 32–34), 2 examined MLR (31, 32), 9 examined lymphocytes (18, 20, 21, 24–26, 32–34), 4 examined monocytes (18, 26, 32, 33), 8 examined HGB (10, 11, 18, 20, 27–29, 34), 4 examined HCT (10, 18, 20), 2 examined RBC (18, 20), 8 examined PLT (18, 20, 24, 25, 29, 32–34), 3 examined PLR (19, 25, 32), 4 examined NLR (21, 25, 31, 32), 3 examined MPV (10, 19, 25), 3 examined PDW (10, 19, 30), 2 examined CRP (30, 32), and 3 examined SIRI (31–33). The NOS assessment demonstrated good overall quality, as detailed in Table 1.

### 3.3 Meta-analysis

#### 3.3.1 Differences in HPs between the BPD and non-BPD groups

Differences in WBC, NEU, MLR, HGB, lymphocytes, monocytes, HCT, RBC, PLT, NLR, PLR, MPV, RDW, PDW, CRP, and SIRI in BPD and non-BPD patients were analyzed in the BPD and non-BPD groups. The results indicated statistical significance for NEU (WMD: 0.89, 95% CI: 0.23, 1.55, *P* = 0.008; Supplementary Figure 1A), HGB (WMD: −8.97, 95% CI: −10.5, −7.45, *P* = 0.001; Supplementary Figure 1B), HCT (WMD: −3.04, 95% CI: −5.68, −0.41, *P* = 0.023; Supplementary Figure 1C), RBC (WMD: −0.31, 95% CI: −0.43, −0.18, *P* = 0.001; Supplementary Figure 1D), PLT (WMD: −24.89, 95% CI: −42.86, −6.91, *P* = 0.007; Supplementary Figure 2A), NLR (WMD: 0.27, 95% CI: 0.11, 0.43, *P* = 0.001; Supplementary Figure 2B), and SIRI (WMD: 1.27, 95% CI: 0.58, 1.97, *P* = 0.001; Supplementary Figure 2C) with *P* < 0.05. Compared to the non-BPD group, the BPD group had elevated NEU, NLR, and SIRI, as well as reduced HGB, HCT, RBC, and PLT (Table 2).

**Table 2:** Differences in HPs levels between the BPD and non-BPD groups.

Parameter	No. of articles	WMD	95% CI	I^2^ (%)	*P*_heterogeneity_	*P* value
WBC	7	1.16	−1.58, 3.9	97.4	*P* < 0.001	0.407
**NEU**	**9**	**0**.**89**	**0.23, 1.55**	**73**.**4**	*P* < 0.001	**0**.**008**
MLR	2	0.02	−0.01, 0.06	53.7		0.227
**HGB**	**8**	**−8**.**97**	**−10.5, −7.45**	**91**.**4**	***P*** **<** **0.001**	**0**.**001**
Lymphocytes	7	−0.36	−0.86, 0.13	83.8	*P* < 0.001	0.147
Monocytes	4	0.05	−0.02, 0.13	2.2		0.164
**HCT**	**3**	**−3**.**04**	**−5.68, −0.41**	**77**.**8**	***P*** **<** **0.001**	**0**.**023**
**RBC**	**2**	**−0**.**31**	**−0.43, −0.18**	**0**		**0**.**001**
**PLT**	**8**	**−24**.**89**	**−42.86, −6.91**	**81**		**0**.**007**
**NLR**	**4**	**0**.**27**	**0.11, 0.43**	**51**.**4**		**0**.**001**
PLR	3	2.15	−3.68, 7.99	53.1		0.386
MPV	3	0.31	−0.41, 1.03	96.3		0.402
RDW	3	1.29	−1.35, 3.93	97.8		0.338
PDW	3	0.46	−0.17, 1.10	83.6		0.155
CRP	2	0.14	−0.02, 0.30	0		0.077
**SIRI**	**3**	**1**.**27**	**0.58, 1.97**	**67**.**5**		**0**.**001**

#### 3.3.2 Correlation analysis between HPs and BPD

Five studies examined the relationship between PLT and BPD, three studies focused on the relationship between HGB and BPD, and another three studies explored the relationship between HCT and BPD. All three sets of analyses employed an REM owing to significant statistical heterogeneity (PLT: *I*^2^ = 84.3%, *P* < 0.0001; HGB: *I*^2^ = 92.2%, *P* < 0.0001; HCT: *I*^2^ = 95.5%, *P* < 0.0001). The results showed that none of these indicators were significantly linked to BPD. For PLT, the OR was 1.0 with a 95% CI of (0.98, 1.03) (*P* > 0.05). For HGB, the OR was 1.38 with a 95% CI of (0.42, 4.49) (*P* > 0.05). For HCT, the OR was 1.33 with a 95% CI of (0.11, 2.77) (*P* > 0.05). Details are presented in Supplementary Figure 3A–C, respectively.

#### 3.3.3 Heterogeneity exploration

To clarify the sources of heterogeneity, a stratified analysis was conducted based on two key clinical variables potentially affecting result consistency: BPD diagnostic criteria and GA, Supplementary Figures 4, 5. Stratification by diagnostic criteria significantly reduced heterogeneity solely for the NEU parameter. The overall analysis indicated high heterogeneity for NEU (*I*^2^ = 73.4%, *P* < 0.01). After stratifying by criteria, heterogeneity markedly decreased in the subgroup employing the “2018 NICHD criteria” (*I*^2^ = 17%, *P* = 0.272) and also declined in the “2001 NICHD criteria” subgroup (*I*^2^ = 40.7%, *P* = 0.134). Conversely, other HPs maintained high heterogeneity post-stratification: HCT (*I*^2^ = 90.1%, *P* < 0.01), HGB (*I*^2^ = 91.4%, *P* < 0.01), lymphocytes (*I*^2^ = 83.8%, *P* < 0.01), PLT (*I*^2^ = 81.0%, *P* < 0.01), and WBC (*I*^2^ = 97.4%, *P* < 0.01). These parameters exhibited persistently high heterogeneity across subgroups defined by the “2018 NICHD,” “2001 NICHD,” and “2019 NRN” criteria. Stratification by GA failed to substantially improve heterogeneity for any parameter, suggesting that GA differences may not be a primary driver of the observed heterogeneity.

Subgroup analysis further revealed that the diagnostic criteria directly influenced the statistical significance of the pooled results. For instance, the WMD for NEU was 2.12 (95% CI: 1.37, 2.88) under the 2018 NICHD criteria and 0.47 (95% CI: −0.07, 1.00) under the 2001 criteria. This discrepancy underscores how divergent conclusions for the same parameter can arise from different standards, potentially leading to conflicting interpretations and hindering evidence synthesis. Consequently, these findings highlight the urgent need for standardized diagnostic criteria in BPD research.

### 3.4 HPs for BPD diagnosis

#### 3.4.1 NLR

Three studies examined the performance of NLR for BPD diagnosis. The results indicated that the pooled sensitivity, specificity, LR+, and LR- were 0.66 (0.60, 0.72), 0.6 (0.54, 0.65), 1.88 (1.1, 3.2), and 0.6 (0.43, 0.84), respectively (Supplementary Figures 6A–E). The ROC-AUC was 0.67 (standard error [SE] = 0.0536), as shown in Supplementary Figure 6F and Table 3.

**Table 3:** Predictive value of HPs on BPD.

Parameters	No. of articles	Sensitivity (95% CI)	Specificity (95% CI)	LR + (95% CI)	LR- (95% CI)	ROC-AUC(SE)
NLR	3	0.66 (0.6,0.72)	0.6 (0.54, 0.65)	1.88 (1.1,3.2)	0.6 (0.43,0.84)	0.6875 (0.0536)
PLT	4	0.51 (0.45,0.57)	0.7 (0.65,0.75)	1.82 (1.29,2.57)	0.65 (0.5,0.85)	0.6745 (0.0670)

#### 3.4.2 PLT

Four studies examined the performance of PLT for BPD diagnosis. The results indicated that the pooled sensitivity, specificity, LR+, and LR− were 0.51 (0.45, 0.57), 0.7 (0.65, 0.75), 1.82 (1.29, 2.57), and 0.65 (0.5, 0.85), respectively (Supplementary Figures 7A–E). The ROC-AUC was 0.6745 (SE = 0.0670), as illustrated in Supplementary Figure 7F and Table 3.

### 3.5 Sensitivity analysis

Sensitivity analyses of WBC, NEU, HGB, lymphocytes, monocytes, HCT, PLT, NLR, PLR, MPV, RDW, PDW, and SIRI produced stable and reliable results, as displayed in Supplementary Figures 8, 9.

### 3.6 Publication bias

A publication bias analysis was performed using funnel plots (Supplementary Figures 10–12) for WBC, NEU, MLR, HGB, lymphocytes, monocytes, HCT, RBC, PLT, NLR, PLR, MPV, RDW, PDW, CRP, and SIRI. The results indicated possible publication bias.

## 4 Discussion

Bronchopulmonary dysplasia (BPD) is a frequent respiratory complication in preterm infants. It is closely linked to higher mortality, long-term lung function impairment, and lifelong effects on children (35–38). Studies have shown that systemic inflammation indicators contribute significantly to many respiratory diseases. The levels of hyperoxia exposure and inflammatory response in infants with BPD vary based on the degree of hypoxia, and the number and function of NEU, monocytes, and lymphocytes may change (39, 40). Therefore, identifying high-risk infants early and intervening promptly are crucial to reduce neonatal mortality and minimizing the incidence of BPD. Hematological tests are routinely performed on preterm infants after birth to assess their health status. Studies have demonstrated that different blood cells significantly contribute to lung inflammation and subsequent injury in preterm infants (21, 41). However, the relationship between HPs and BPD remains unclear. Further studies are urgently needed to elucidate the potential associations and clinical implications. The current study investigated the association of 16 HPs with moderate-to-severe BPD using a meta-analysis.

According to the study results, the BPD group exhibited markedly higher levels of NEU, NLR, and SIRI compared to the non-BPD group. The *p*-value for CRP was 0.077, approaching statistical significance and suggesting a potential upward trend in the BPD group that requires further validation. These findings support the **inflammation-driven hypothesis of BPD pathogenesis** and align with earlier studies indicating that BPD's pathological process is rooted in an imbalance between pro-inflammatory and anti-inflammatory factors that triggers chronic lung tissue damage (42).

To elaborate, the inflammatory response contributes to BPD development through two key pathways. First, it directly damages lung tissue. Second, it exacerbates pulmonary pathological changes by releasing cytokines and growth factors (43, 44). This effect is particularly pronounced in preterm infants whose immature lungs are highly sensitive to inflammatory factors, making them more susceptible to inflammatory injury and further promoting BPD onset (45, 46). **As the main effector cells of innate immunity, NEU can directly aggravate lung tissue damage by releasing ROS and proteases** (47). This explains why elevated NEU in the BPD group further reinforces the inflammation-driven mechanism of BPD (9, 47).

Notably, composite inflammatory indicators, such as NLR and SIRI, are more sensitive to systemic inflammatory imbalances than single markers (48). While the BPD group showed statistically significant differences in NLR, the limited predictive value, evidenced by a pooled effect size of WMD, may stem from inconsistent detection time points and GA across studies. From a **clinical perspective**, this highlights two key implications. First, the study's results reinforce the clinical relevance of targeting inflammatory pathways for BPD prevention or intervention. Second, future research must standardize study designs (e.g., unified detection time points and stratified analyses by GA) to clarify the clinical threshold of NLR and validate its utility in predicting BPD. This will address current limitations and enhance the translational value of NLR in clinical practice.

The current study indicated that the BPD group had significantly lower HGB, HCT, RBC, and PLT than the non-BPD group. While levels of PLT, HGB, and HCT were significantly lower in the BPD group, their pooled ORs did not show a significant correlational association with the risk of developing BPD. These findings might reflect deficiencies in oxygenation function in children with BPD. Chronic hypoxia stimulates an increase in erythropoietin (EPO) production (49). EPO is a key factor in regulating erythropoiesis. Its compensatory elevation may suggest an adaptive response to hypoxia (48). However, persistent hypoxia and the release of inflammatory factors can inhibit or accelerate the destruction of erythropoiesis. This exacerbates anemia and hypoxia, creating a vicious cycle. Furthermore, lower HGB levels compromise the blood's oxygen-carrying capacity (50, 51), leading to an inadequate supply of oxygen to tissues. This, in turn, increases heart rate and cardiac output, which may induce organ dysfunction (52). Preterm infants are prone to anemia due to frequent blood collection, inadequate nutrition, and other factors. Anemia can decrease the tissue oxygen supply, which increases lactate levels through anaerobic metabolism. This exacerbates pulmonary vascular remodeling and fibrosis, contributing to the progression of BPD (53, 54). Although we observed elevated NEU, NLR, and SIRI, as well as reduced HGB, HCT, RBC, and PLT, our correlation analysis indicated no significant correlation between HGB and BPD. Thus, anemia may be a concomitant phenomenon of BPD. Future studies must incorporate stratified analyses of oxygen therapy strategies, transfusion history, and other clinical factors to elucidate the complex relationship between anemia and BPD. For instance, investigating the correlation of anemia with the severity of BPD or examining how different oxygen therapy strategies affect anemia and BPD progression could provide new insights for clinical intervention.

This meta-analysis found no significant correlation of PLT with the risk of BPD. Although PLT contributes significantly to inflammation and vascular remodeling by releasing growth factors and chemokines, this study revealed no significant association between PLT and BPD. This finding might be related to the following factors: (i) PLT dynamics are influenced by various confounders (e.g., infection in preterm infants, history of transfusion, or maternal pregnancy complications), which may obscure its specific association with BPD; (ii) PLT function (rather than number) may be more directly involved in BPD pathology (e.g., PLT activation-mediated pulmonary microvascular injury); however, existing studies are limited to PLT count and lack functional data; (iii) The time window for PLT detection may affect the results. PLT was detected in the early postnatal period (e.g., within 72 h) in some studies. However, the chronic inflammatory process of BPD may not significantly affect PLT kinetics until later. Further diagnostic performance analyses indicated that PLT had limited predictive power for BPD. Its pooled sensitivity, specificity, and ROC-AUC were 0.51 (95% CI: 0.45–0.57), 0.70 (95% CI: 0.65–0.75), and 0.67 (95% CI: 0.0670), respectively, suggesting low clinical value as a biomarker alone. Nevertheless, the specificity (0.70) and LR+ (1.82) of PLT indicated that it could help exclude some non-BPD cases (e.g., when combined with other indicators). Its low sensitivity (0.51) and high LR− (0.65) suggested that relying on PLT alone resulted in a higher risk of false negatives. An ROC-AUC value of 0.67 confirmed that its predictive efficacy was insufficient, with a significant gap compared to the composite inflammation indicators (NLR, SIRI, etc.) reported in previous studies (AUC > 0.70 in general) (52). These results suggest that PLT has a limited role in predicting BPD as a single parameter, but future studies may explore combining PLT with inflammation or hypoxia indicators to improve the discriminative ability of comprehensive models.

The current study has the following strengths. First, it is the first meta-analysis to systematically assess the correlation between 16 HPs and moderate-to-severe BPD. This approach overcame the limitations of previous single-indicator or small-sample studies, potentially improving the level of evidence by integrating multicenter data. Second, this meta-analysis innovatively analyzed the composite inflammation indicators (NLR and SIRI) in combination with classical inflammation indicators (NEU). This approach potentially verified the centrality of inflammation in the pathological mechanism of BPD and provided a new direction for future biomarker studies. Third, the meta-analysis identified a significant correlation between BPD and lower HGB levels, which is consistent with the pathophysiological model of erythropoietic suppression under chronic hypoxia. Although anemia could theoretically exacerbate impaired lung development by limiting oxygen delivery, the relationship is more likely the result of shared underlying pathological processes, such as inflammation and oxidative stress. Current evidence is insufficient to establish HGB reduction as an independent factor directly impairing alveologenesis or pulmonary vascular maturation. Instead, its clinical significance may lie in signifying synergistic systemic hypoxia aggravation or serving as a surrogate marker of disease severity. Future mechanistic studies are warranted to elucidate the role of anemia in lung development.

Several key limitations warrant consideration when interpreting the associations between HPs and BPD. First, significant heterogeneity was observed in nearly all inter-study comparisons. Despite employing an REM and conducting exploratory analyses for BPD diagnostic criteria and GA, the sources of heterogeneity were not fully resolved. Potential contributors include differences in baseline birth weight and maternal pregnancy conditions, inconsistent timing of HPs assessments, and insufficient exclusion of confounding effects from neonatal infections. Incomplete reporting in the primary studies precluded further stratification to clarify these factors and may have undermined the reliability of the pooled estimates. Second, the limited number of available studies globally resulted in a small overall sample size susceptible to small-study effects and a general lack of critical clinical data, such as threshold values for HPs. This gap impedes the translation of findings into actionable clinical decision-making tools. Furthermore, the small number of included studies and inconsistent timing of blood sampling across studies precluded a meta-regression analysis comparing early vs. late time points, considering both analytical constraints and clinical applicability. Third, restricting the analysis to English-language publications may have introduced selection bias by omitting relevant non-English studies and potentially overemphasizing positive results. Finally, the confounding effect of neonatal transfusion therapy requires particular attention. Transfusion of blood products alters levels of parameters such as HGB and PLT. Inconsistent recording of transfusion timing, product type, and the interval between transfusion and blood sampling across the primary studies, coupled with a lack of statistical adjustment for this confounder, may obscure the true relationship between parameter abnormalities and transfusion-induced fluctuations on BPD risk.

## 5 Conclusion

This meta-analysis suggests an association between BPD and several HPs, including NEU, PLT, HGB, HCT, RBC, NLR, and SIRI. However, the effectiveness of NLR and PLT as predictive biomarkers for BPD appears limited. These findings imply that early monitoring and targeted management of inflammation-associated parameters, such as NEU and SIRI, might offer potential avenues for improving BPD outcomes in preterm infants. Nevertheless, given the outlined limitations, the pooled results primarily indicate associations rather than confirm causality and should not be directly adopted as a basis for clinical intervention. Interpretation requires caution to avoid overgeneralization.

To further elucidate the relationship between HPs and BPD and facilitate clinical translation, three key directions for future research are proposed: (i) Conduct large-sample, multicenter, high-quality case-control studies employing uniform diagnostic criteria, such as the 2018 NICHD definition, to minimize heterogeneity from criterion differences; (ii) Mandate detailed documentation of transfusion-related specifics in study designs (e.g., transfusion timing, product type, and transfusion-to-blood collection interval) and incorporate serial measurements of HPs to enable dose-response analyses for establishing critical thresholds and validating the preventive efficacy of parameter modulation through clinical intervention trials; (iii) Employ multi-language search strategies in subsequent meta-analyses to include non-English and gray literature (e.g., conference abstracts), complemented by subgroup (stratified by GA and transfusion status) and sensitivity analyses to verify the robustness of the associations. This will ultimately provide more reliable evidence for BPD prediction and intervention in neonates.

## Data Availability

The original contributions presented in the study are included in the article/Supplementary Material, further inquiries can be directed to the corresponding author.
