# Supplementary material for: Hematological parameters of bronchopulmonary dysplasia in preterm infants: a meta-analysis
Source: Front Pediatr. 2025 Nov 17;13:1657314. doi: 10.3389/fped.2025.1657314 (PMC12665771; doi:10.3389/fped.2025.1657314)
Supplement: Supplementary file 1 [file Supplementaryfile1.docx]

**Supplementary Materials**

**Supplementary Table S1** Search strategy

|  | **Ovid Pubmed** |
| --- | --- |
| 1 | Bronchopulmonary Dysplasia[MeSH Terms] |
| 2 | (Bronchopulmonary Dysplasia[Title/Abstract]) OR (lung dysplasia[Title/Abstract]) |
| 3 | (Bronchopulmonary Dysplasia[MeSH Terms]) OR ((Bronchopulmonary Dysplasia[Title/Abstract]) OR (lung dysplasia[Title/Abstract])) |
| 4 | Blood Platelets[MeSH Terms] |
| 5 | (Thrombocytes[Title/Abstract]) OR (Thrombocyte[Title/Abstract]) OR (Platelets[Title/Abstract]) OR (Platelet[Title/Abstract]) OR (thrombocyte[Title/Abstract]) OR (hematological parameters[Title/Abstract]) OR (Blood Platelets[Title/Abstract]) OR (Zinc[Title/Abstract]) OR (B12[Title/Abstract]) OR (Iron[Title/Abstract]) OR (Folic Acid[Title/Abstract]) OR (Ferritin[Title/Abstract]) OR (Hemoglobin[Title/Abstract]) OR (Hematocrit[Title/Abstract]) OR (neutrophil-to-lymphocyte ratio[Title/Abstract]) OR (NLR[Title/Abstract]) OR (platelet-to-lymphocyte ratio[Title/Abstract]) OR (PLR[Title/Abstract]) OR (lymphocyte-to-monocyte ratio[Title/Abstract]) OR (LMR[Title/Abstract]) OR (platelet count[Title/Abstract]) OR (PLT[Title/Abstract]) OR (mean platelet volume[Title/Abstract]) OR (platelet mass index[Title/Abstract]) OR (white blood cell count[Title/Abstract]) OR (WBC[Title/Abstract])) OR (red blood cell count[Title/Abstract]) OR (RBC[Title/Abstract]) OR (hemoglobin[Title/Abstract]) OR (HgB[Title/Abstract]) OR (erythrocyte sedimentation rate[Title/Abstract]) OR (ESR[Title/Abstract]) OR (mean cell hemoglobin concentration[Title/Abstract]) OR (MCHC[Title/Abstract]) OR (mean cell volume[Title/Abstract]) OR (MCV[Title/Abstract]) OR (mean cell hemoglobin[Title/Abstract]) OR (MCH[Title/Abstract])] |
| 6 | (Blood Platelets[MeSH Terms]) OR ((Thrombocytes[Title/Abstract]) OR (Thrombocyte[Title/Abstract]) OR (Platelets[Title/Abstract])) OR (Platelet[Title/Abstract])) OR (thrombocyte[Title/Abstract])) OR (hematological parameters[Title/Abstract])) OR (Blood Platelets[Title/Abstract])) OR (Zinc[Title/Abstract])) OR (B12[Title/Abstract])) OR (Iron[Title/Abstract])) OR (Folic Acid[Title/Abstract])) OR (Ferritin[Title/Abstract])) OR (Hemoglobin[Title/Abstract])) OR (Hematocrit[Title/Abstract])) OR (neutrophil-to-lymphocyte ratio[Title/Abstract])) OR (NLR[Title/Abstract])) OR (platelet-to-lymphocyte ratio[Title/Abstract])) OR (PLR[Title/Abstract])) OR (lymphocyte-to-monocyte ratio[Title/Abstract])) OR (LMR[Title/Abstract])) OR (platelet count[Title/Abstract])) OR (PLT[Title/Abstract])) OR (mean platelet volume[Title/Abstract])) OR (platelet mass index[Title/Abstract])) OR (white blood cell count[Title/Abstract])) OR (WBC[Title/Abstract])) OR (red blood cell count[Title/Abstract])) OR (RBC[Title/Abstract])) OR (hemoglobin[Title/Abstract])) OR (HgB[Title/Abstract])) OR (erythrocyte sedimentation rate[Title/Abstract])) OR (ESR[Title/Abstract])) OR (mean cell hemoglobin concentration[Title/Abstract])) OR (MCHC[Title/Abstract])) OR (mean cell volume[Title/Abstract])) OR (MCV[Title/Abstract])) OR (mean cell hemoglobin[Title/Abstract])) OR (MCH[Title/Abstract])) |
| 7 | ((Bronchopulmonary Dysplasia[MeSH Terms]) OR ((Bronchopulmonary Dysplasia[Title/Abstract]) OR (lung dysplasia[Title/Abstract]))) AND ((Blood Platelets[MeSH Terms]) OR ((Thrombocytes[Title/Abstract]) OR (Thrombocyte[Title/Abstract])) OR (Platelets[Title/Abstract])) OR (Platelet[Title/Abstract])) OR (thrombocyte[Title/Abstract])) OR (hematological parameters[Title/Abstract])) OR (Blood Platelets[Title/Abstract])) OR (Zinc[Title/Abstract])) OR (B12[Title/Abstract])) OR (Iron[Title/Abstract])) OR (Folic Acid[Title/Abstract])) OR (Ferritin[Title/Abstract])) OR (Hemoglobin[Title/Abstract])) OR (Hematocrit[Title/Abstract])) OR (neutrophil-to-lymphocyte ratio[Title/Abstract])) OR (NLR[Title/Abstract])) OR (platelet-to-lymphocyte ratio[Title/Abstract])) OR (PLR[Title/Abstract])) OR (lymphocyte-to-monocyte ratio[Title/Abstract])) OR (LMR[Title/Abstract])) OR (platelet count[Title/Abstract])) OR (PLT[Title/Abstract])) OR (mean platelet volume[Title/Abstract])) OR (platelet mass index[Title/Abstract])) OR (white blood cell count[Title/Abstract])) OR (WBC[Title/Abstract])) OR (red blood cell count[Title/Abstract])) OR (RBC[Title/Abstract])) OR (hemoglobin[Title/Abstract])) OR (HgB[Title/Abstract])) OR (erythrocyte sedimentation rate[Title/Abstract])) OR (ESR[Title/Abstract])) OR (mean cell hemoglobin concentration[Title/Abstract])) OR (MCHC[Title/Abstract])) OR (mean cell volume[Title/Abstract])) OR (MCV[Title/Abstract])) OR (mean cell hemoglobin[Title/Abstract])) OR (MCH[Title/Abstract]))) |

|  | **Embase <1974 to 2025 April 10>** |
| --- | --- |
| 1 | 'lung dysplasia'/exp |
| 2 | 'bronchopulmonary dysplasia':ab,ti OR 'lung dysplasia':ab,ti |
| 3 | #1 OR #2 |
| 4 | 'thrombocyte'/exp OR 'hematological parameters'/exp |
| 5 | 'blood platelets':ab,ti OR thrombocytes:ab,ti OR thrombocyte:ab,ti OR platelets:ab,ti OR platelet:ab,ti OR 'hematological parameters':ab,ti OR zinc:ab,ti OR cyanocobalamin:ab,ti OR iron:ab,ti OR 'folic acid':ab,ti OR ferritin:ab,ti OR hematocrit:ab,ti OR nlr:ab,ti OR 'neutrophil lymphocyte ratio':ab,ti OR 'platelet-to-lymphocyte ratio':ab,ti OR plr:ab,ti OR 'lymphocyte-to-monocyte ratio':ab,ti OR lmr:ab,ti OR 'platelet count':ab,ti OR plt:ab,ti OR 'mean platelet volume':ab,ti OR 'platelet mass index':ab,ti OR 'white blood cell count':ab,ti OR wbc:ab,ti OR 'red blood cell count':ab,ti OR erythrocyte:ab,ti OR hemoglobin:ab,ti OR hgb:ab,ti OR 'erythrocyte sedimentation rate':ab,ti OR esr:ab,ti OR 'mean cell hemoglobin concentration':ab,ti OR mchc:ab,ti OR 'mean cell volume':ab,ti OR mcv:ab,ti OR 'mean cell hemoglobin':ab,ti OR mch:ab,ti |
| 6 | #4 OR #5 |
| 7 | #3 AND #6 |

|  | **Web of Science** |
| --- | --- |
| 1 | Bronchopulmonary Dysplasia (Topic) or lung dysplasia (Topic) |
| 2 | Blood Platelets (Topic) or Thrombocytes (Topic) or Thrombocyte (Topic) or Platelets (Topic) or Platelet (Topic) or hematological parameters (Topic) or Zinc (Topic) or B12 (Topic) or Iron (Topic) or Folic Acid (Topic) or Ferritin (Topic) or Hemoglobin (Topic) or Hematocrit (Topic) or NLR (Topic) or neutrophil-to-lymphocyte ratio (Topic) or platelet-to-lymphocyte ratio (Topic) or PLR (Topic) or lymphocyte-to-monocyte ratio (Topic) or LMR (Topic) or platelet count (Topic) or PLT (Topic) or mean platelet volume (Topic) or platelet mass index (Topic) or white blood cell count (Topic) or WBC (Topic) or red blood cell count (Topic) or RBC (Topic) or hemoglobin (Topic) or Hgb (Topic) or erythrocyte sedimentation rate (Topic) or ESR (Topic) or mean cell hemoglobin concentration (Topic) or MCHC (Topic) or mean cell volume (Topic) or MCV (Topic) or mean cell hemoglobin (Topic) or MCH (Topic) |
| 3 | #1 AND #2 |

|  | **Cochrane Library** |
| --- | --- |
| 1 | MeSH descriptor: [Bronchopulmonary Dysplasia] explode all trees |
| 2 | (Bronchopulmonary Dysplasia):ti,ab,kw OR (lung dysplasia):ti,ab,kw |
| 3 | MeSH descriptor: [Blood Platelets] explode all trees |
| 4 | (Blood Platelets):ti,ab,kw OR (Thrombocytes):ti,ab,kw OR (Thrombocyte):ti,ab,kw OR (Platelets):ti,ab,kw OR (Platelet):ti,ab,kw |
| 5 | (hematological parameters):ti,ab,kw OR (Zinc):ti,ab,kw OR (B12):ti,ab,kw OR (Iron):ti,ab,kw OR (Folic Acid):ti,ab,kw |
| 6 | (Ferritin):ti,ab,kw OR (Hemoglobin):ti,ab,kw OR (Hematocrit):ti,ab,kw OR (NLR):ti,ab,kw OR (neutrophil-to-lymphocyte ratio):ti,ab,kw |
| 7 | (platelet-to-lymphocyte ratio):ti,ab,kw OR (PLR):ti,ab,kw OR (lymphocyte-to-monocyte ratio):ti,ab,kw OR (LMR):ti,ab,kw OR (platelet count):ti,ab,kw |
| 8 | (PLT):ti,ab,kw OR (mean platelet volume):ti,ab,kw OR (platelet mass index):ti,ab,kw OR (white blood cell count):ti,ab,kw OR (WBC):ti,ab,kw |
| 9 | (red blood cell count):ti,ab,kw OR (RBC):ti,ab,kw OR (hemoglobin):ti,ab,kw OR (HgB):ti,ab,kw OR (erythrocyte sedimentation rate):ti,ab,kw |
| 10 | (ESR):ti,ab,kw OR (mean cell hemoglobin concentration):ti,ab,kw OR (MCHC):ti,ab,kw OR (mean cell volume):ti,ab,kw OR (MCV):ti,ab,kw |
| 11 | (mean cell hemoglobin):ti,ab,kw OR (MCH):ti,ab,kw |
| 12 | #1 or #2 |
| 13 | #3 or #4 or #5 or #6 or #7 or #8 or #9 or #10 or #11 |
| 14 | #12 and #13 |

**Supplementary Figures**

**
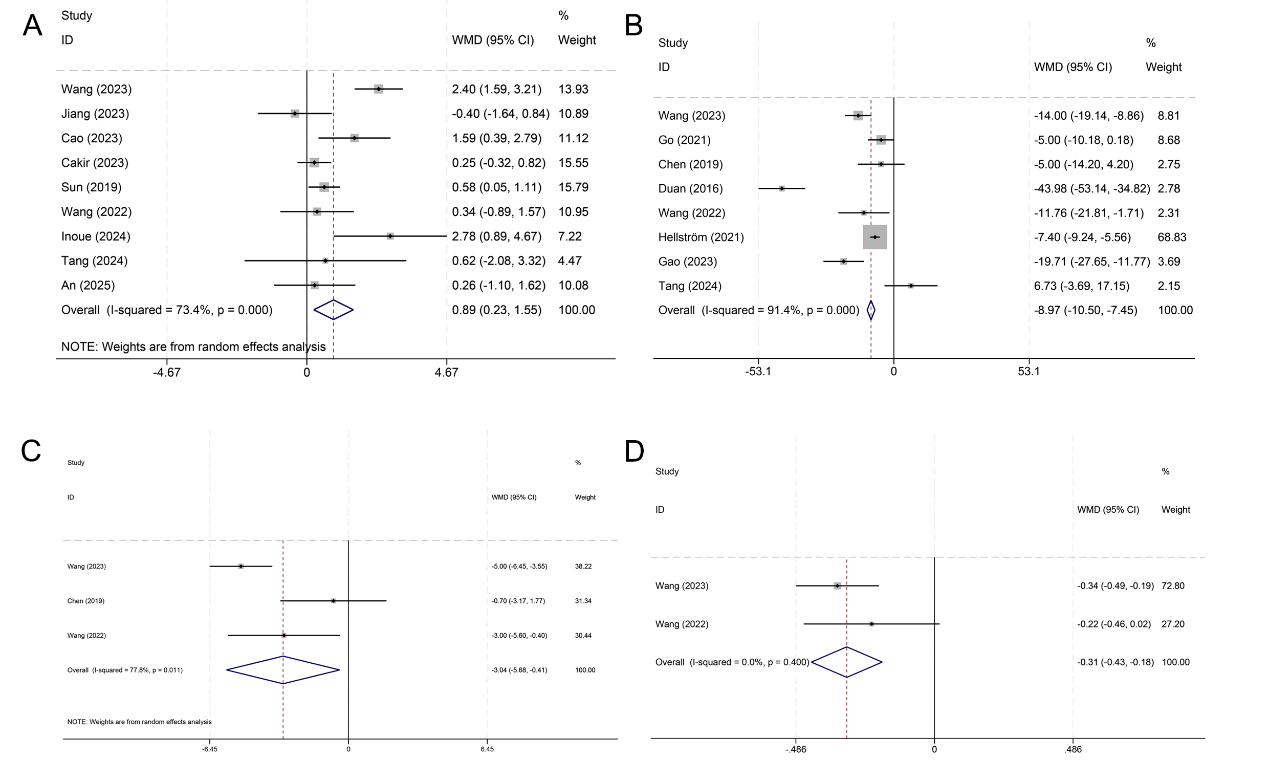
**

**Supplementary Figure 1** The forest plot of hp differences between the BPD group and the non-BPD group. (A) Neutrophils; (B) HGB; (C) HCT; (D) RBC.


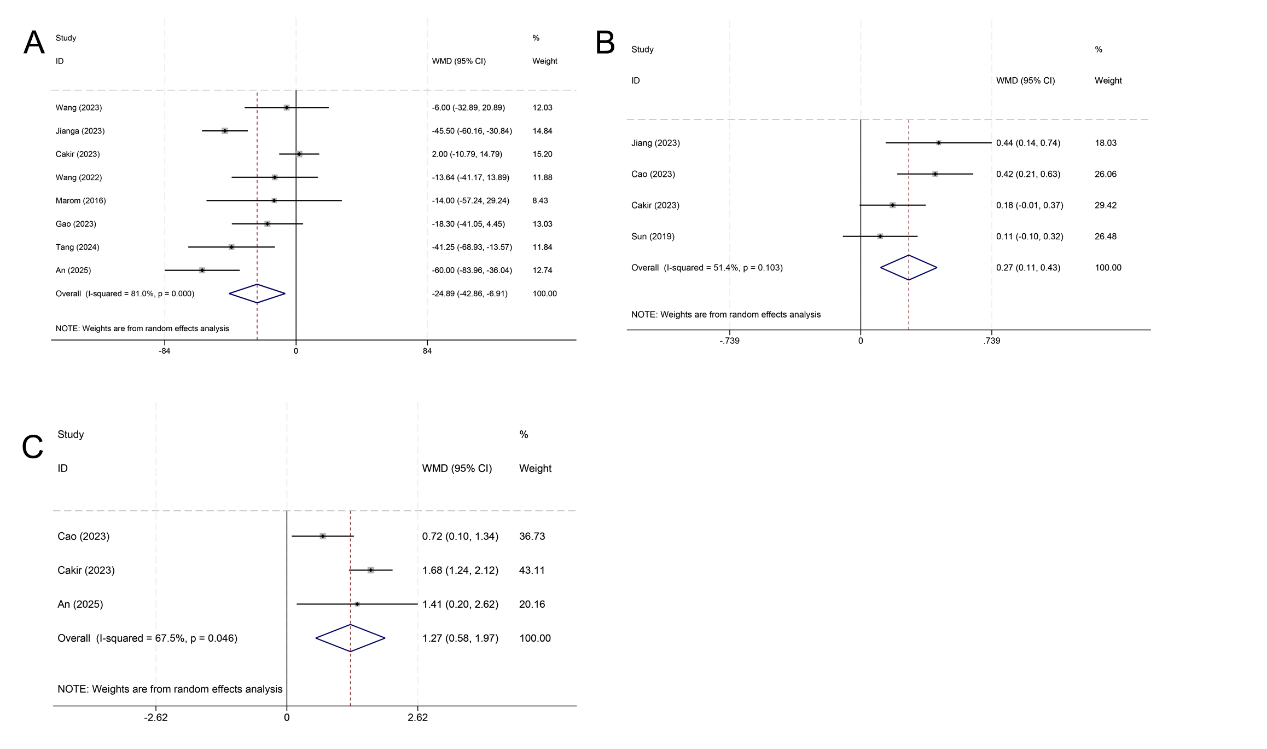


**Supplementary Figure** **2** The forest plot of hp differences between the BPD group and the non-BPD group. (A) PLT; (B) NLR; (C) SIRI.


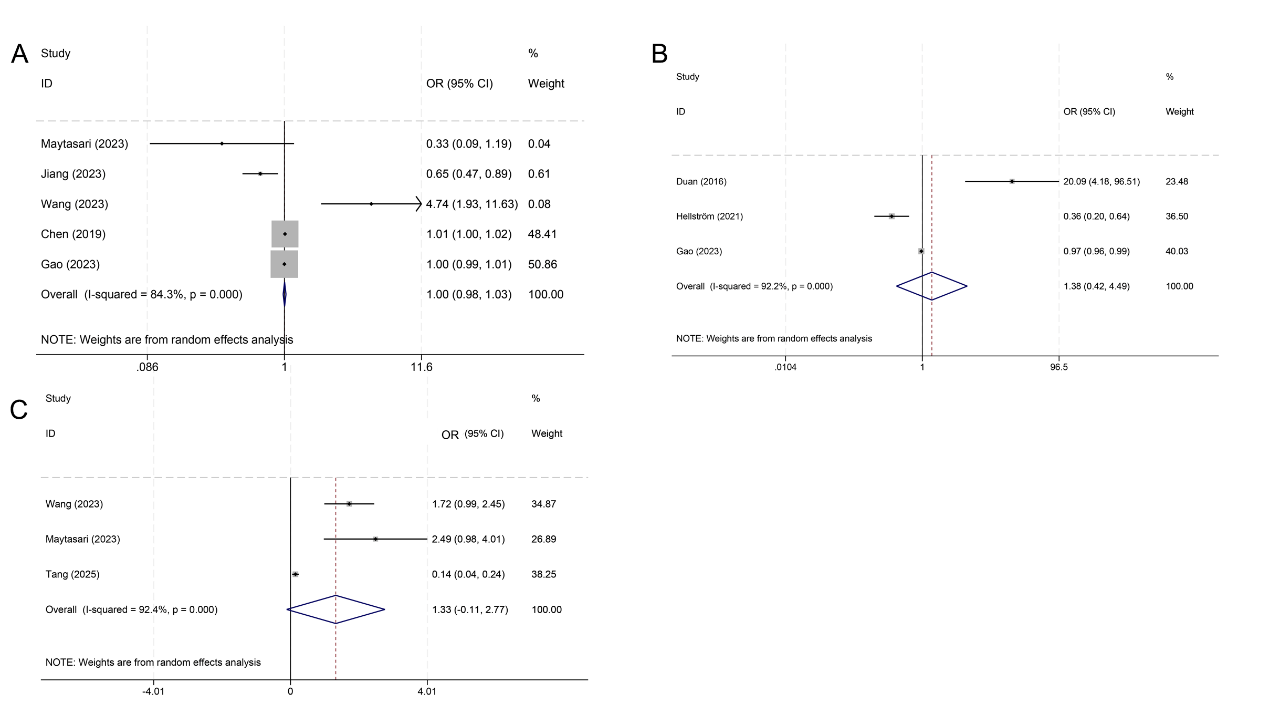


**Supplementary Figure 3** The forest plot of the correlation analysis between hp and BPD. (A) PLT; (B) HB; (C) HCT.


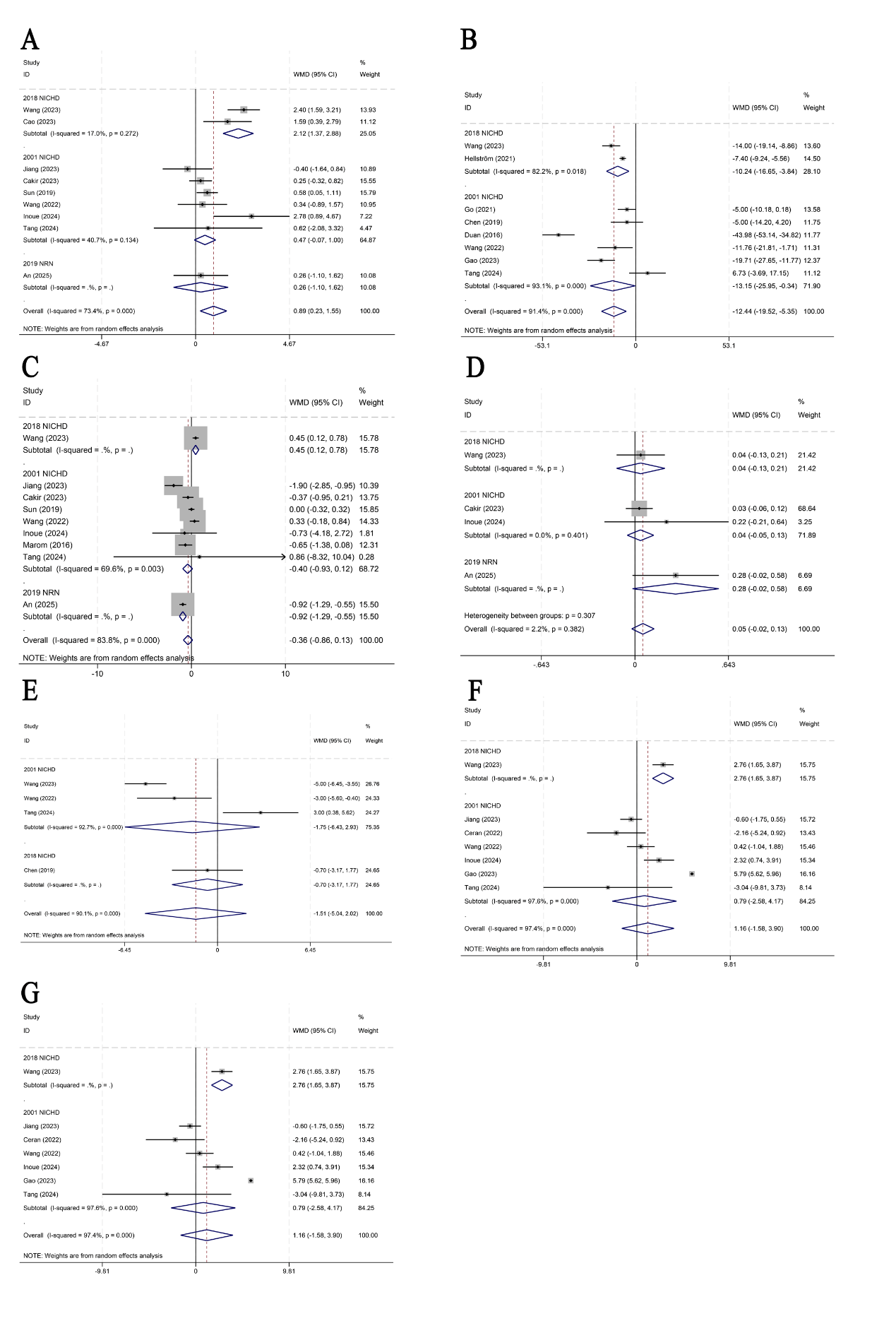


**Supplementary Figure** **4** Heterogeneity Assessment of HPs versus BP According to Diagnostic Criteria. (A) NEU; (B) HGB; (C) Lymphocytes; (D) Monocytes; (E)HCT; (F)WBC; (G)PLT

**
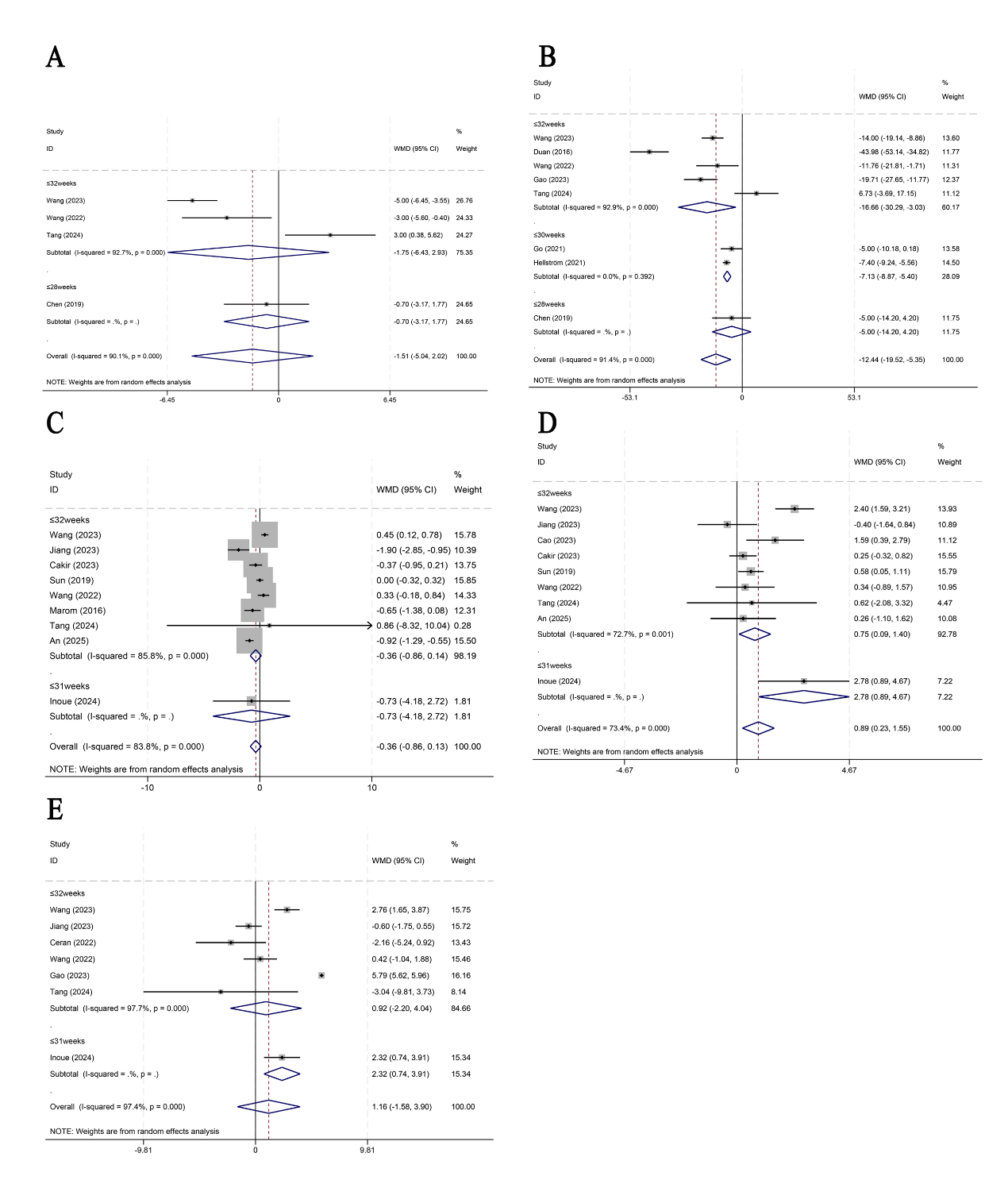
**

**Supplementary Figure** **5** Exploration of Heterogeneity in the Association Between HPs and BPD Stratified by Gestational Age. (A)HCT; (B) HGB; (C) Lymphocytes; (D) NEU; (E) WBC.


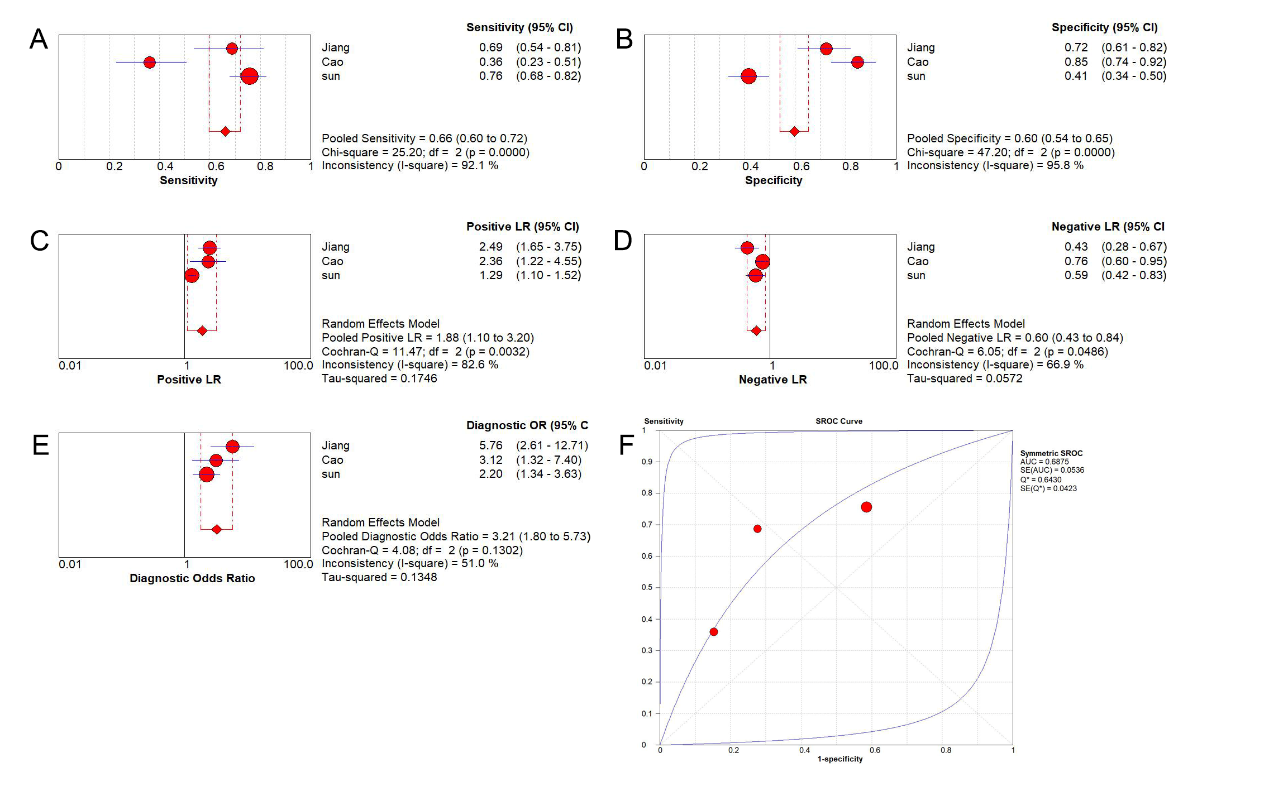


**Supplementary Figure** **6** The analysis results of NLR for the diagnosis of BPD. (A) Sensitivity; (B) Specificity; (C) Positive likelihood ratio; (D) Negative likelihood ratio; (E) Diagnostic odds; (F) ROC-AUC.


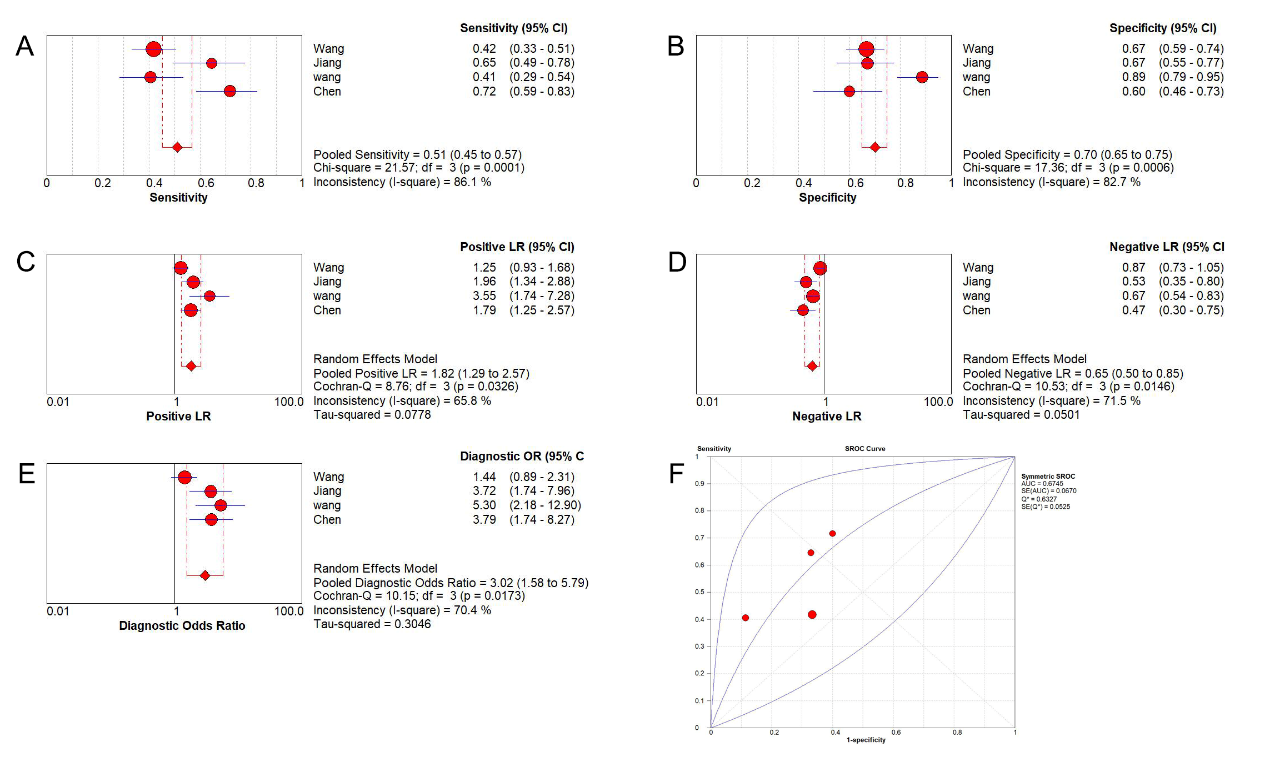


**Supplementary Figure** 7 The analysis results of PLT for the diagnosis of BPD. (A) Sensitivity; (B) Specificity; (C) Positive likelihood ratio; (D) Negative likelihood ratio; (E) Diagnostic odds; (F) ROC-AUC.


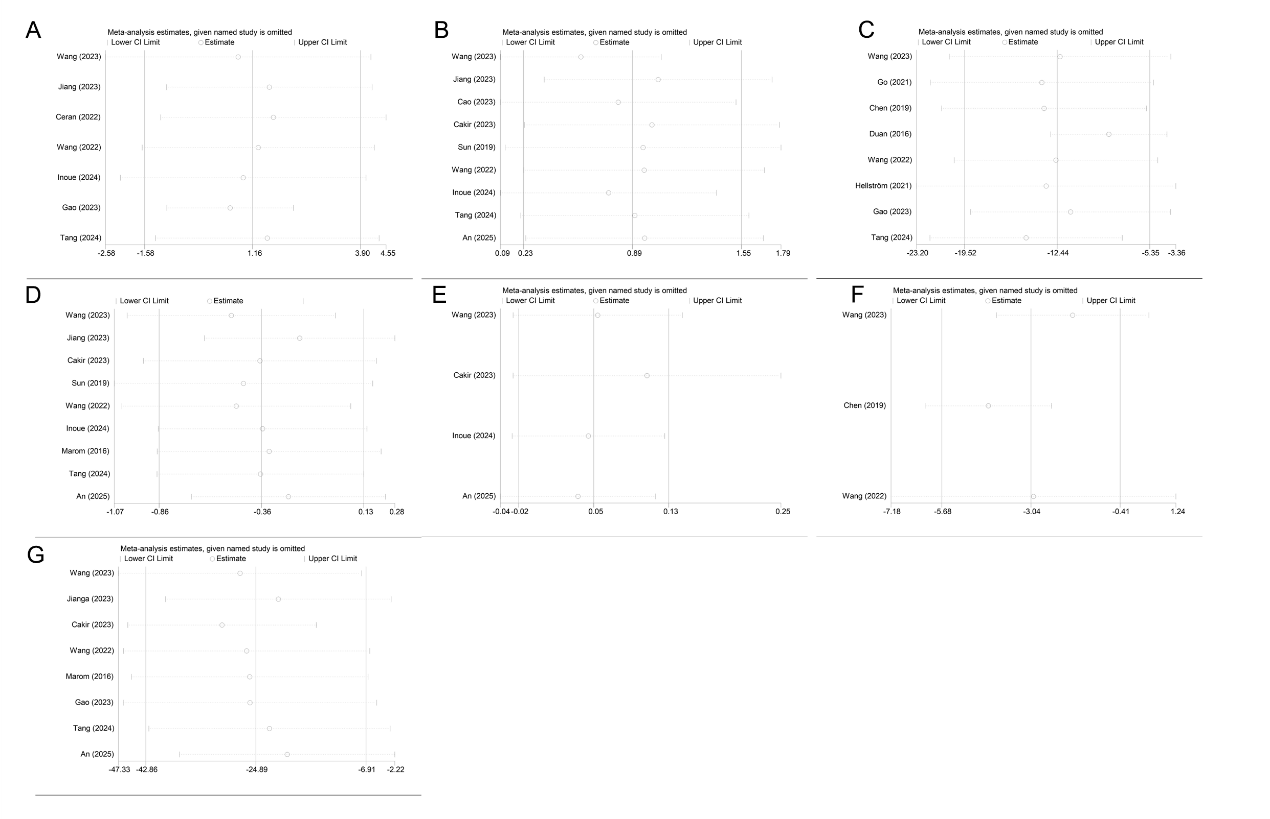


**Supplementary Figure** **8** Sensitivity analysis of the results. (A) WBC; (B) Neutrophils; (C) HGB; (D) Lymphocytes; (E) Monocytes; (F) HCT; (G) PLT.


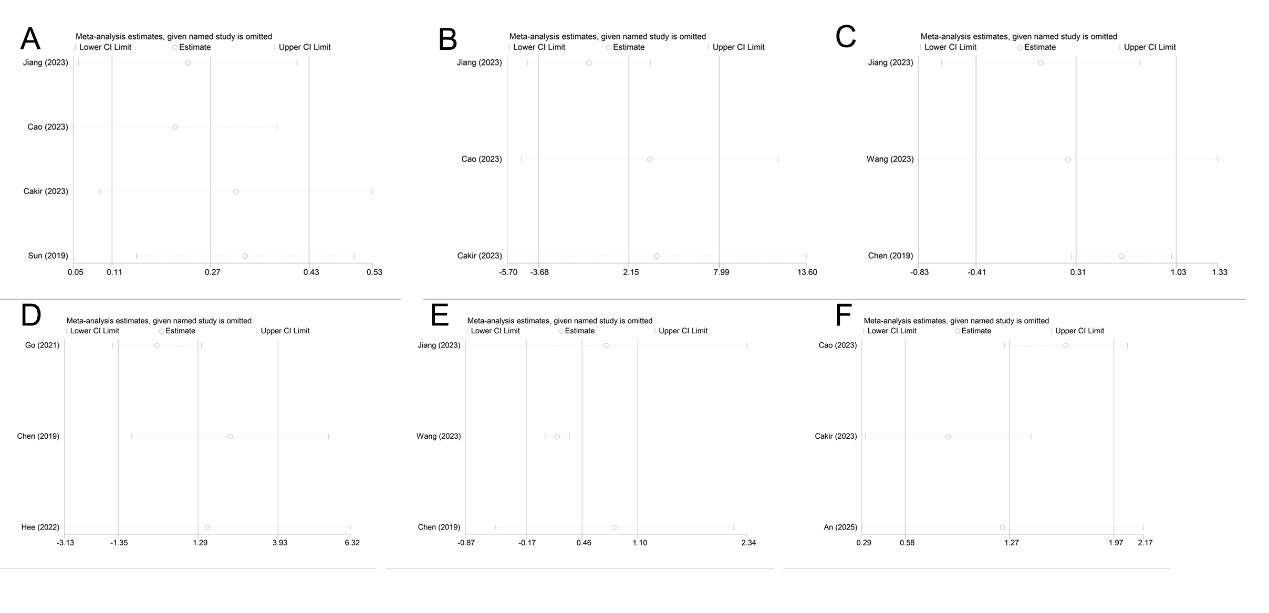


**Supplementary Figure** **9** Sensitivity analysis of the results. (A) NLR; (B) PLR; (C) MPV; (D) RDW; (E) PDW; (F) SIRI.


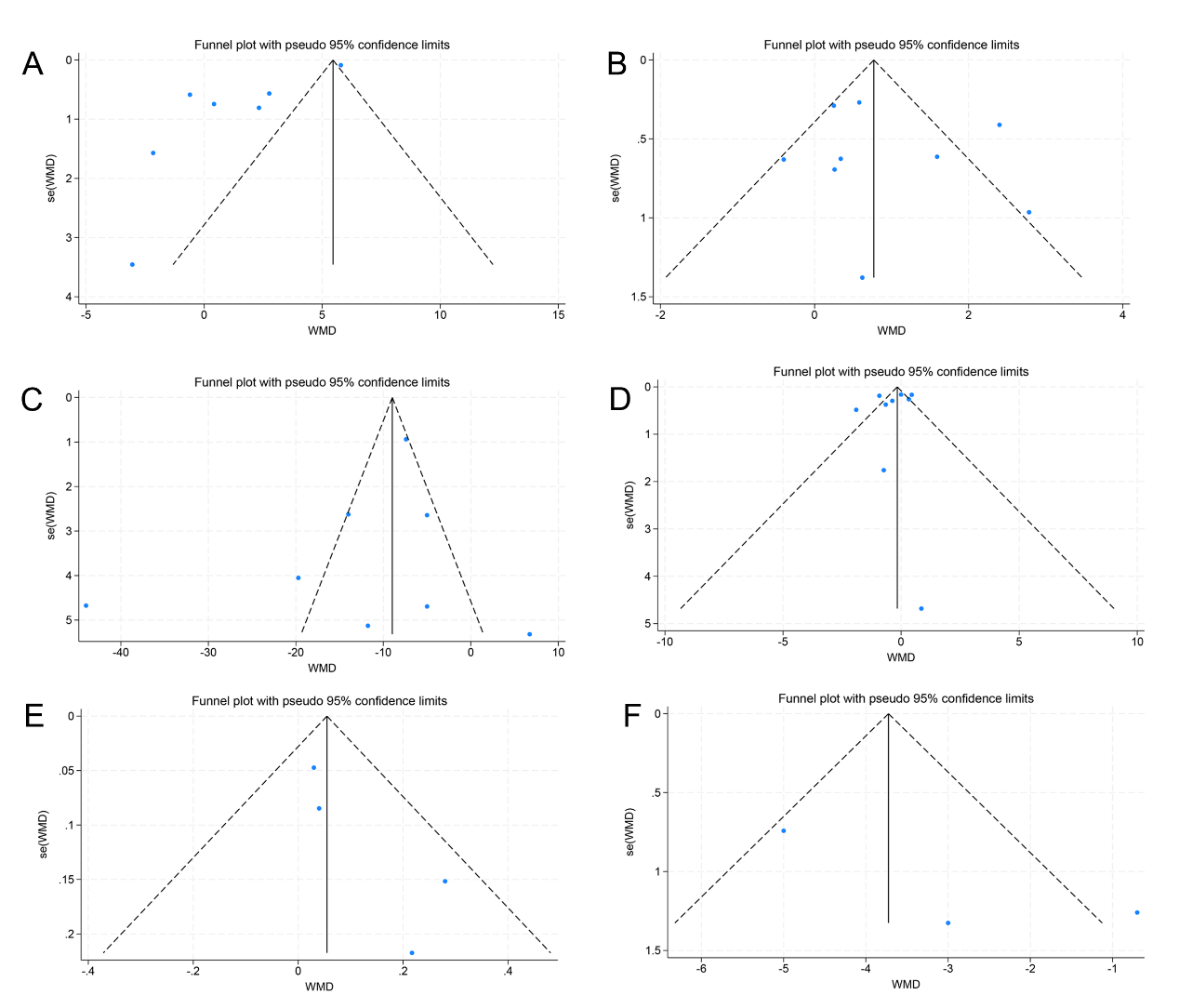


**Supplementary Figure** **10** Funnel plot of the results. (A)WBC;(B) Neutrophils; (C) HGB;(D) Lymphocytes;(E) Monocytes;(F) HCT.


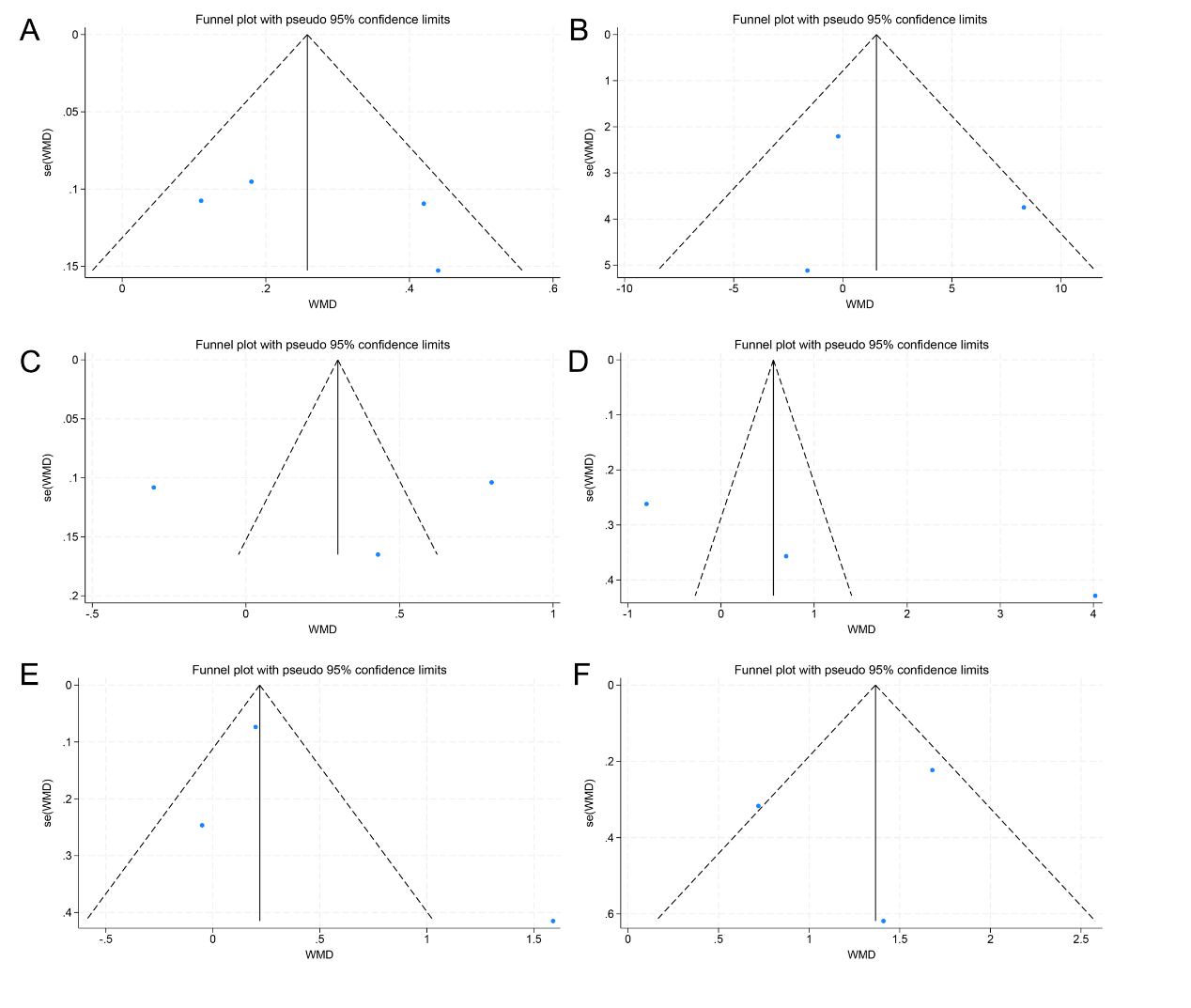


**Supplementary Figure** **11** Funnel plot of the results. (A) NLR; (B) PLR; (C) MPV; (D) RDW; (E) PDW; (F) SIRI.


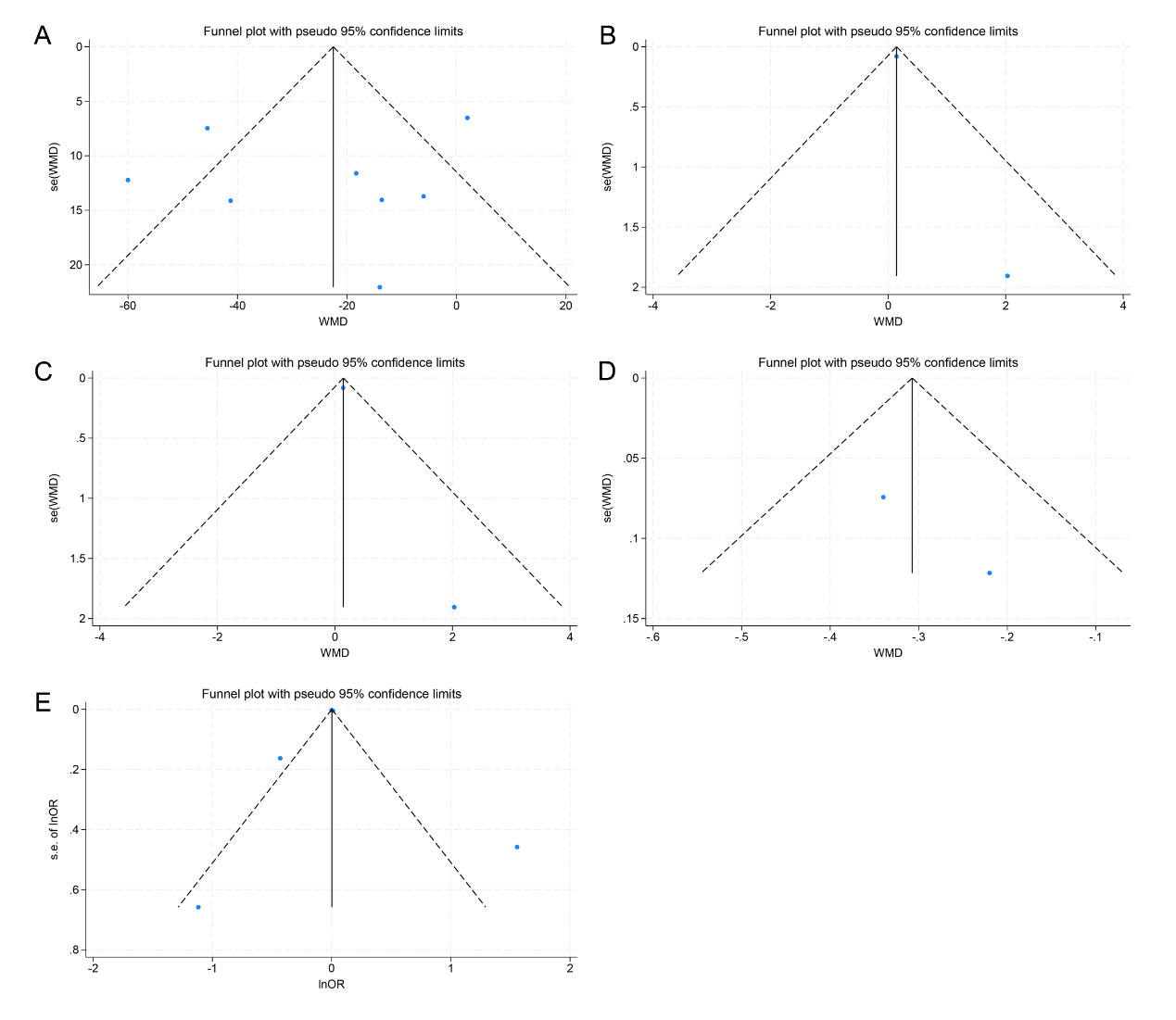


**Supplementary Figure** **12** Funnel plot of the results. (A) PLT; (B) CRP; (C) MLR; (D) RBC; (E) PLT (Correlation analysis).
